# The Relationship Between Perceived Social Support and Learning Engagement Among Chinese Vocational Students: A Moderated Chain Mediation Model

**DOI:** 10.3390/bs16030426

**Published:** 2026-03-16

**Authors:** Zhanqi Xu, Yuanqing He

**Affiliations:** College of Educational Science, Anhui Normal University, Wuhu 241000, China; rachelxuzq@163.com

**Keywords:** perceived social support, learning engagement, emotional resilience, mindfulness, depression, vocational students

## Abstract

Learning engagement is essential in vocational education that can help in students’ development and equip them academically and professionally. The present study aimed to elucidate the dynamic interplay of perceived social support and engagement in learning among Chinese vocational students, examining a chain of mediation of emotional resilience and mindfulness and the moderating role of depression. A questionnaire was administered to 1545 Chinese vocational students. The evidence from this study suggested that (1) the perceived social support had a significant positive direct effect on learning engagement; (2) emotional resilience and mindfulness are chain mediators linking perceived social support to learning engagement; and (3) there was a significant moderating effect of depression on the mindfulness-to-engagement pathway. In particular, the facilitative effects of mindfulness on engagement in learning in cases of increased depression levels were comparably undermined. These results are useful in explaining the psychological processes underlying the effects of perceived social support on learning engagement among students in Chinese vocational colleges. Beyond the theoretical value, the evidence helps vocational educators develop more precise interventions for both learning support and mental well-being.

## 1. Introduction

The General Secretary Xi Jinping explicitly stated at the 2024 National Education Conference that there is a pressing need to “build a vocational education system that integrates vocational training with general education and combines industry with education, and vigorously cultivate master craftsmen, skilled artisans, and high-skilled talents”, charting the future course for vocational education in China. Under this guidance, higher vocational education, as the principal means of training high-quality technical skills, is experiencing significant expansion and receiving growing emphasis within China’s higher education system. The quality of excellence in nurturing talent has become the focus of attention in both academia and society (Jing et al., 2025; Shi & Hao, 2025). The level of learning engagement provides a primary metric for assessing the quality of students’ learning processes. It typically refers to the sustained effort, emotional involvement, and concentration demonstrated by individuals in learning activities. The implications of learning engagement’s level extend beyond scholastic achievement to encompass both mental health and career development (Greco et al., 2025; M. Li et al., 2022; Nouwen et al., 2022; Wong et al., 2024). Vocational college students tend to be under more specific pressure than their counterparts in standard higher learning institutions and in aspects of academic growth, career planning, and social identity (Jing et al., 2025; L. Wang et al., 2022). Thus, their degree of learning activity is more prone to the overlapping effect of the social context and personal psychological conditions (Keijzer et al., 2022; Sureda-García et al., 2021). Due to academic success and a social stereotype, vocational college students can face an increase in psychological stress and a lack of confidence in further development (Z.-H. Zhao et al., 2023). This necessitates a systematic exploration of the factors and processes of their interaction affecting the learning engagement of students in vocational colleges, which must have enormous theoretical and practical value in relation to the better organization of the general quality of vocational studies in China.

Social support is one such contextual variable among the alleviating factors in facilitating positive learning behaviors among students. Conservation of Resources (COR) theory claims that social support is a crucial external resource; support provided by relatives, friends, and others makes a private person feel cared for and understood, helping them cope with stress and gain ever-growing psychological capital (Hobfoll et al., 2018; Y. Song & He, 2025). Social support commonly comprises two primary types: that which is objectively available and that which is perceived. Compared to objective social support, perceived social support by persons is more effective in predicting learning results and psychological adaptation (Tian et al., 2025; Wu et al., 2023). Perceived social support focuses on an individual’s subjective experience, whether a person is comprehended, nurtured, and embraced (Zimet et al., 1988). This experience, per se, is a significant psychological resource, and it has the potential to affect learning engagement in a number of psychological ways (Cohen & Wills, 1985).

Prior studies have provided evidence for a strong link between perceived social support and learning engagement (Gutiérrez et al., 2017; Luan et al., 2023; Sun et al., 2023). Still, the question of the psychological mechanism, according to which the perceived social support is translated into an internal motivation, contributing to enhancing learning engagement, warrants future inquiry. Recent empirical studies have discovered that emotional resilience is significantly and positively associated with both learning engagement and learning performance (González-Yubero et al., 2025; Namaziandost et al., 2023). It is reasonable to deduce that a high level of emotional resilience could be used as a psychological resource, increasing the motivation of students towards the learning process and supporting their involvement in the learning process. In addition, mindfulness has been established to enhance the self-regulation skills of students and strongly boost their involvement in learning (Hammill et al., 2023). However, little is known about the sequential roles of emotional resilience and mindfulness in the process of perceived social support and learning engagement and whether depression moderates these relationships. Addressing these gaps is essential for developing targeted interventions to support this vocational population. Consequently, in this paper, the population being targeted is a group of Chinese vocational college students, processing emotional resilience and mindfulness as the intermediate variables that are used to investigate their contributions in the predictive process from perceived social support to learning engagement. Moreover, the moderating influence of depression is examined to improve the knowledge base of psychological processes that underlie the effects of learning engagement by vocational college learners.

### 1.1. Perceived Social Support and Learning Engagement

Perceived social support is the personal belief that care, understanding, and assistance are available from one’s social network (e.g., relatives, friends, and teachers) and, ultimately, the emotional feeling of being valued and supported (Barrera, 1986; Haber et al., 2007; McDonald et al., 2022). It is stated that perceived social support constitutes a significant psychological resource for individuals, according to the COR (Cohen & McKay, 2020). It may offer emotional solace and informational advice to students so that they can handle academic stress and dampen stress, hence triggering motivation and encouraging engagement (Bakker et al., 2014; Baltacı & Karataş, 2015; Sevari et al., 2020). Prior studies have demonstrated that multi-source social support is positively and significantly related to academic adaptation, learning motivation, and the achievement of university students (Kong & You, 2013; J. Zhao & Qin, 2021) and acts as a pivotal environmental element in facilitating energetic learning behaviors (Zhu et al., 2025). But in contrast with objective social support, aimed at the availability of external resources, perceived social support reflects more the subjective estimate of the quality of social relations a person has (Zimet et al., 1988). Students who have increased perceived social support have higher learning enthusiasm and engagement (Prananto et al., 2025). In the case of vocational students, being acknowledged and supported in the immediate surrounding environment helps the student feel part of it and boosts their self-worth. Such positive psychological experience may then be translated into vitality, commitment, and focus during learning, and it will ultimately manifest itself into higher degrees of learning participation and successes in schoolwork (Schaufeli et al., 2002; Gutiérrez et al., 2017; Tao et al., 2022; Guo et al., 2025).

Moreover, through the lens of Self-Determination Theory (SDT), a supportive external environment satisfies the basic psychological need for relatedness in the person involved. This leads to an improvement in intrinsic motivation, which then increases the active engagement in learning processes (Ryan & Deci, 2020; Y. Wang et al., 2024). As students feel understood and respected by significant others throughout the learning process, they will tend to perceive learning activities as the result of their own choices rather than a reaction to external pressure. Such cognition helps increase commitment to learning (Xu et al., 2024).

Studies across a variety of populations have validated the overall positive correlation that exists in the dynamic of perceived social support and learning engagement (Rautanen et al., 2022; Sun et al., 2023; X. Zhang & Qian, 2024). Perceived social support in the stabilizing condition helps reduce students’ threat assessment of academic challenges, thereby promoting sustained learning engagement. Thus, the understanding and consciousness of social support can be used to assist students in minimizing their stress, diminishing such negative emotional reactions caused by stress, and motivating them to experience learning challenges to better increase their involvement in learning.

As indicated by the analysis above, this research is guided by the following hypothesis:

**H1.**  *Perceived social support exerts a significant positive predictive effect on learning engagement among Chinese vocational students.*

### 1.2. The Mediating Role of Emotional Resilience

Emotional resilience is considered to be the capability of someone to behave and recuperate positive emotional states in response to stresses and setbacks, a resource in psychological adaptation that can be molded and cultivated (Tugade & Fredrickson, 2004; Snijders et al., 2018), which is not only crucial for mental well-being but also essential to the maintenance of task-focused learning behaviors (B. Li et al., 2022; Galve-González et al., 2025). The COR indicates that the higher the amount of psychological resources that an individual has, the less likely that resources will be depleted in stressful conditions (Hobfoll et al., 2018). In other words, within the framework of conceptualizing social support as an indispensable psychological resource, a person can be ensured with a safe environment to better manage their emotions, which increases their emotional resilience (Taylor, 2011). Empirical evidence suggests that perceived social support and emotional resilience share a significant positive association, where such correlations are stable (Cao et al., 2024; Guo et al., 2025). Increased perceived social support can greatly decrease distress and improve psychological resiliency and buffering capacity (Cohen & Wills, 1985; C. Song et al., 2023), thus alleviating the impact of other unfavorable variables on physical and mental well-being (Cao et al., 2024).

The Broaden-and-Build Theory (BBT) of positive emotions notes that positive feelings can extend the range of thought and action of a person, and in the long term, their accumulation may create permanent personal resources, including psychological resilience (Fredrickson, 2001, 2004). When students feel supported, the positive feelings of pleasure, gratitude, and hope that they experience when dealing with difficulties make them more open and flexible in their thinking, which partly builds and empowers their own emotional resilience (Ding et al., 2017). Emotional resilience, as a crucial personal resource, assists students in having a positive attitude towards academic failures, as they can be reframed as a growth opportunity and not a menace. It decreases negative emotions, which enables them to maintain high learning motivation and involvement (Beaumont et al., 2023; Carmona-Halty et al., 2021; Y.-D. Yang et al., 2024). Therefore, this study posits the following hypothesis:

**H2.**  *Emotional resilience mediates the relationship between perceived social support and learning engagement among Chinese vocational students.*

### 1.3. The Mediating Role of Mindfulness

Mindfulness is usually conceptualized as a cultivatable ability to be fully present and receptive to moment-to-moment experiences with openness and acceptance. Its fundamental features are a concentration on current activities and non-judgmental acceptance of internal and external experience (Brown & Ryan, 2003; Kabat-Zinn, 2001; Kabat-Zinn et al., 1985). Being an inherent mental and emotional control skill, mindfulness has been reaffirmed to correlate with numerous beneficial psychological and environmental outcomes, such as greater psychological prosperity and reduced depressive and apprehension states (Aldbyani et al., 2025; Bamber & Kraenzle Schneider, 2016). This psychological trait is important and encourages students to concentrate on their learning and control their emotions (Zenner et al., 2014). By providing emotional security in learning situations, positive supportive experiences help reduce excessive worry and rumination when confronted with an academic task, paving the way for the development of stable mindful awareness (Chang et al., 2015; Creswell, 2017; Gu et al., 2015). That is, perceived social support offers a positive mental condition for developing the mindfulness capacity of an individual. Mindfulness practice will allow students to regulate and direct their attention and experience less distraction due to inappropriate thoughts and shift students’ mental energies towards the task at hand, learning, as compared to ruminating on thoughts of judgment or perceptions of failures (Molina et al., 2025; Ostermann et al., 2022). At the same time, the tolerant mindset promoted by mindfulness could assist students in approaching learning challenges and an overall negative mood with a clearer mind without emotional depletion or anxiety, thus maintaining their learning energy and commitment (Reginald, 2023). Moreover, mindfulness can improve the sense of flourishing and meaning in life of a person by promoting cognitive mechanisms such as positive reappraisal (Y. Wang et al., 2023), assisting students in experiencing the joy and importance of the learning process.

Thus, perceived social support can facilitate learning engagement indirectly by fostering individual mindfulness qualities. Derived from our analysis, the following hypothesis is offered in this research:

**H3.**  *Mindfulness mediates the relationship between perceived social support and learning engagement among Chinese vocational students.*

### 1.4. The Chain Mediating Role of Emotional Resilience and Mindfulness

In terms of psychological function, the social support that students feel helps them first stabilize their emotional state when they are stressed by learning; they will not always be trapped in negative feelings (Dahlem et al., 1991). This way, students can have more psychological space and cognitive resources, focus on current learning tasks, and are willing to take on new things with openness and acceptance, and engagement in learning increases.

The social support people feel is the key psychological resource that can help them accumulate emotional reserves and enhance their emotional resilience. When emotional resilience improves, people will feel more positive emotions, and the scale of cognitive and behavioral reactions will be expanded, making it easier to develop a stable mindfulness experience (Jaiswal et al., 2018; Keng et al., 2013; Ozonder Unal & Ordu, 2023; Y.-Y. Tang et al., 2015). On this basis, mindfulness promotes focus and involvement in learning activities through reducing distractions and ruminative thinking (Indriaswuri et al., 2023; Kuroda et al., 2022; Liu et al., 2022). From this, perceived social support is an effective contributor to learning engagement through the consecutive psychological pathways of emotional resilience and mindfulness. According to these analyses, this study suggests the following hypothesis:

**H4.**  *Emotional resilience and mindfulness form a chained mediation pathway linking perceived social support to learning engagement among Chinese vocational students.*

### 1.5. The Moderating Role of Depression in Linking Mindfulness to Learning Engagement

Depression, as a common negative emotional state, is frequently accompanied by a lack of motivation; the inability to concentrate, negatively influencing one’s cognitive bias; and a serious inhibitory influence on learning behaviors (Zhou et al., 2024). Students in vocational colleges are at high risk of being depressed as they have many pressures, including college work and employment (Gao et al., 2024). After all, learners who are more depressed tend to have a disadvantage in the domain of engaged learning behaviors and perseverance in terms of school grades (Awadalla et al., 2020). Even though it is postulated that mindfulness has a buffering effect on negative behaviors (Hofmann et al., 2010; Vorontsova-Wenger et al., 2021), it is not necessarily equally effective in all situations (Firth et al., 2019).

As a person’s perceived value of learning activities and perceived control is dramatically lower, the positive psychological state of such a person cannot be effectively converted into concrete behavior (Pekrun, 2006). The main symptoms of depression include anhedonia and a loss of motivation that exert a direct adverse effect on the perceived value of learning and the perceived control by students (Gotlib & Joormann, 2010). This means that although a student who is highly depressed has some degree of mindfulness capacity, they might not demonstrate high learning engagement owing to a deficiency of intrinsic learning drive. This implies that depression can moderate the relationship between attitude to learning and mindfulness. Particularly, at lower levels of depression, mindfulness might be of greater use to students at risk to stay focused and consistently engaged, and at higher levels of depression, the potential positive influence of mindfulness might be limited. According to the analysis above, this research suggests the following hypothesis that needs to be tested:

**H5.**  *Depression has a moderating effect on the relationship between mindfulness and learning engagement.*

**Figure 1:**
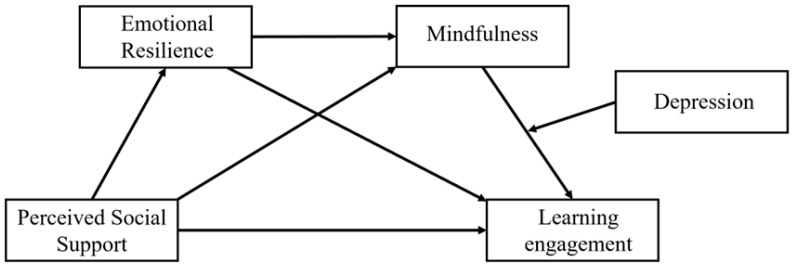
Hypothesized model.

To explore these complex dynamics, this research builds a moderated chain mediation model (Figure 1) in which emotional resilience and mindfulness sequentially mediate the relationship between perceived social support and learning engagement, and depression moderates the mindfulness–engagement pathway among Chinese vocational college students.

### 1.6. Aims

Making a synthesis of the literature provided above, it should be noted that perceived social support functions as a positive explanatory variable of the learning engagement of students, as mentioned to a certain extent. However, a more detailed study still needs to be done. First, current research tends to treat social support as a relatively static external condition, and there is inadequate investigation of the area of individual subjective experience in terms of the consequences of perceived social support in mediating learning engagement using internal psychological processes. Second, whereas earlier studies have already confirmed the role of emotional resilience or mindfulness individually in learning behaviors, no specific attention has been paid to the possibility of a sequential interaction between the two factors in the framework of one model. This is especially so in the case of the vocational college student population, where further empirical investigation is needed to substantiate this relationship.

In addition, the prevailing studies of the role of mindfulness in learning behaviors tend to assume that it has a universal promoting effect, and not much attention is paid to the fact that differences in the effects of mindfulness on learning may arise due to individual emotional problems. Indeed, the phenomenon of depression being a comparatively frequent emotional challenge in vocational college students has already been shown to hinder the motivation to learn and learning behaviors (Awadalla et al., 2020). It is against this background that there is a need to further trace the possibility of a role played by depression in terms of moderating the actual role of mindfulness in learning situations.

On this basis, in this research project, Chinese vocational college students are the research respondents, and distinguishing between perceived and objective social support, this research project evolves into a chain mediation model that includes emotional resilience and mindfulness. It also takes the moderating effect of depression in the association between mindfulness and involvement in learning.

## 2. Materials and Methods

### 2.1. Participants

This research adopted a convenience sampling technique to select participants that are vocational college students in higher vocational institutions in Anhui Province. This study was done in the form of an online integrated questionnaire. The instructions included the aim of this research, the requirements to complete the questionnaire, and the principle of anonymity and were read to the respondents, and they gave their informed consent to continue with the questionnaire. There were 2081 questionnaires distributed. Upon the removal of invalid questionnaires that were not taken seriously or took an unusually short amount of time to complete, 1545 valid questionnaires were acquired, leading to an effective response rate of 74.24%. Participants ranged in age from 17 to 23 years (M = 19.27; SD = 1.27). The mean duration taken by the survey participants to answer the questionnaire was about 9 min, and none of them took any longer than the time given. Table 1 displays the demographic characteristics of the participants. This research underwent ethical review and obtained approval from the Ethics Committee of Anhui Normal University.

### 2.2. Measures

#### 2.2.1. Perceived Social Support Scale

The perceived social support scale, originally developed by Zimet et al. (1988) and later adapted by Jiang (1999), was used in this study. This scale consists of three dimensions, among which are Family Support, Friend Support, and Significant Other Support. Each dimension includes 4 items, leading to a total of 12 items. The answers were graded on a 7-point Likert scale, ranging from 1 (strongly disagree) to 7 (strongly agree). They were summed up, and the greater the sum, the greater the perception of social support. The scale showed high structural validity and internal consistency between the Chinese student groups (X. Yang & Han, 2021; Z.-H. Zhao et al., 2023). In the current analysis, Cronbach’s alpha on this scale was 0.95.

#### 2.2.2. Emotional Resilience Scale

The Adolescent Emotional Resilience Questionnaire, which was built and developed by M. Zhang and Lu (2010) on the basis of emotional resilience theory, was employed. The scale has two dimensions as it consists of Positive Emotional Capacity and Emotion Recovery Capacity with 11 items. Items were rated on a scale ranging from 1 (very inconsistent) to 6 (very consistent). All items were brought together to form the total scores, and the higher the score, the more emotional resilience one holds. The Cronbach coefficient for the questionnaire was 0.91 in this study.

#### 2.2.3. Mindful Attention Awareness Scale

The Mindful Attention Awareness Scale, originally developed by Brown and Ryan (2003) and later revised for the Chinese context by Chen et al. (2012), was employed. It has a unidimensional scale of 15 items. Each of the items was rated using a 6-point Likert scale, ranging from 1 (almost always) to 6 (rarely). A higher value of the sum of the scores reflects a more advanced level of mindfulness, i.e., an increased trait-level ability to be present, mindful and attentive in everyday life. The Cronbach’s alpha coefficient of this scale in the current study was 0.93.

#### 2.2.4. Depression Scale

The Patient Health Questionnaire-4 (PHQ-4) was used, which was designed by Kroenke et al. (2009). It is a short psychological health assessment tool with the aim of assessing anxiety and depression. Being a simplified variant of the longer PHQ-9 scale (Kroenke et al., 2009), it includes 4 items that are oriented to assess depressive and anxiety symptoms over the last two weeks. The answers are noted on a 4-point Likert scale ranging from 0 (not at all) to 3 (nearly every day). The Cronbach’s α of this scale in the current study was 0.90.

#### 2.2.5. Learning Engagement Scale

The Utrecht Work Engagement Scale for Students (UWES-S-9), which was constructed by Schaufeli et al. (2002), was used. It mainly examines how much energy, dedication, and absorption one has in terms of learning processes, which represents a form of good experience in the course of learning in a particular situation. The scale was divided into three subscales, namely, energy, dedication, and absorption, consisting of 9 items. The answers were then documented on a Likert scale out of 7, with 0 meaning never and 6 meaning always. An increased composite score means an increased amount of learning involvement. The scale was also observed to be rather reliable and valid among a Chinese student population (X. Li & Huang, 2010). In the current analysis, internal consistency for the scale was assessed with Cronbach’s α, which yielded a value of 0.96.

### 2.3. Data Analysis

Data were statistically analyzed using SPSS 27.0 and Mplus 8.3. First, descriptive statistics and correlation analyses were carried out on core variables measured in this research, and Harman’s single-factor test and the unmeasured latent variable (UMLV) technique were adopted to conduct common method bias (D. Tang & Wen, 2020). Subsequently, to address the limitation of measurement error and evaluate the overall fit of the proposed theoretical framework, we conducted structural equation modeling (SEM) with latent variables using Mplus 8.3. Then, the mediating effects and the moderated chain mediation model were tested using the PROCESS macro (v3.5.3) developed by Hayes (2017). Specifically, PROCESS Model 6 was used to verify the chain mediating effect of emotional resilience and mindfulness in the relationship between perceived social support and learning engagement. PROCESS Model 87 was used to probe the moderating effect of depression on the relationship between mindfulness and learning engagement. All analyses controlled for gender as a covariate. The sampling frequency was set at 5000 times to test a 95% confidence interval and assess the significance of the effect (i.e., whether the interval excluded 0).

## 3. Results

### 3.1. Common Method Bias Test

Since this research utilized self-report questionnaires in data collection, common method bias could be a problem that needs to be considered. Several procedural and statistical controls were used to reduce this bias during the implementation of data collection, which included: the anonymity of the administration of the questionnaires to resolve evaluation apprehension; the clear instruction given to the participants that they should respond to the questionnaires in a manner that expressed only their actual feelings, to reduce social desirability bias; and the use of an attention check item and reverse-coded items in the questionnaire to increase data authenticity and reliability. Based on these procedural controls, Harman’s single-factor test was later used to statistically evaluate the existence of significant common method variance. All items of the research variables underwent a non-rotated exploratory factor analysis. The findings showed that there seven factors were extracted whose eigenvalues were above 1. The former contributed to variance at 38.02%, which is less than the critical figure of 40%. To further assess common method bias, we employed the unmeasured latent variable (UMLV) technique by introducing a common method factor into a bifactor model. The model fit indices did not improve significantly with the addition of this factor (CFI and TLI increased by <0.01, and RMSEA and SRMR decreased by <0.05), suggesting that the systematic variance due to common methods was minimal (Wen et al., 2018). These results support the conclusion that there is no significant common method bias.

### 3.2. Descriptive Statistics and Correlational

The means, standard deviations, and correlation matrix for the main study variables are presented in Table 2. The results showed that perceived social support was significantly positively associated with emotional resilience, mindfulness, and learning engagement and significantly inversely related to depression. Both emotional resilience and mindfulness were markedly and directly proportional to learning engagement and significantly inversely associated with depression. Depression was significantly inversely correlated with learning engagement (*ps* < 0.001).

### 3.3. Structural Validation and Measurement Model

Before testing the hypothesized relationships, we conducted structural equation modeling (SEM) using Mplus 8.3 to examine the overall fit and the distinctiveness of the key constructs: perceived social support, emotional resilience, mindfulness, depression, and learning engagement. To improve model parsimony and the ratio of variable to sample size, item parceling was employed for the perceived social support scale (X_p1, X_p2, X_p3). The measurement model showed the following fit indices: χ^2^(850) = 6338.955; *p* < 0.001; CFI = 0.883; TLI = 0.876; RMSEA = 0.065 (90% CI [0.063, 0.066]).

While the CFI and TLI values were slightly below the conventional threshold of 0.90, the RMSEA indicated acceptable fit. Given the complexity of the model (43 items measuring five constructs) and the large sample size (*N* = 1545), χ^2^ is inflated, and this can negatively influence the magnitude of incremental fit indices like the CFI and TLI. In contrast, the RMSEA, which is less sensitive to sample size in this context, suggested a reasonable approximation to the data. Therefore, considering the model’s complexity and the pattern of fit indices collectively, the measurement model was considered sufficiently robust for subsequent path analysis (Hu & Bentler, 1999; Marsh et al., 2004).

To further verify discriminant validity, we compared this hypothesized five-factor model with a single-factor model (where all items loaded on one latent construct). The results showed that the five-factor model fit the data significantly better than the single-factor model (Δχ^2^ = 14,596.52, Δ*df* = 11, *p* < 0.001), confirming that the five key constructs are statistically distinct.

### 3.4. Chain Mediation Effect of Emotional Resilience and Mindfulness

**Figure 2:**
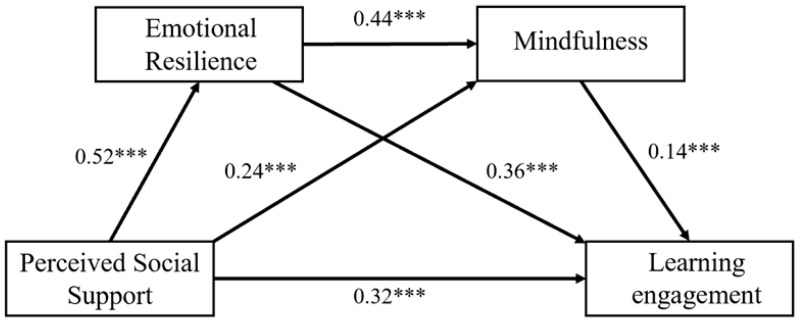
Chain mediation model. *** *p* < 0.001.

To test Hypotheses H2, H3, and H4, a chain mediation analysis was done on Model 6 of the SPSS PROCESS macro (Hayes, 2017). Perceived social support was entered as the independent variable (X), emotional resilience as the first mediator (M1), mindfulness as the second mediator (M2), and learning engagement as the dependent variable (Y) while controlling for gender. The testing of the effects was done using 5000 bootstrap resamples. The results of the regression analysis are summarized in Table 3, with the path coefficients provided in Figure 2.

Step 1: The perceived social support effect on emotional resilience was explored. The findings demonstrated that perceived social support exerted a significant and positive predictive effect on emotional resilience (β = 0.52, *p* < 0.001). Step 2: Perceived social support and emotional resilience were entered into a regression model as predictors of mindfulness. After controlling for emotional resilience, perceived social support remained a significant positive predictor of mindfulness. Perceived social support was also found to predict mindfulness significantly in the post-control group, after adjusting for emotional resilience (β = 0.24, *p* < 0.001). Simultaneously, emotional resilience also significantly and positively predicted mindfulness (β = 0.44, *p* < 0.001). Step 3: The synergistic effects of perceived social support, emotional resilience, and mindfulness on learning engagement were examined. After controlling for emotional resilience and mindfulness, the direct effect of perceived social support on learning engagement remained statistically robust (β = 0.32, *p* < 0.001). Both emotional resilience (β = 0.36, *p* < 0.001) and mindfulness (β = 0.14, *p* < 0.001) also demonstrated a significant capacity to forecast higher levels of learning engagement.

The specific details regarding the total effect, direct effect, indirect effects, and confidence intervals for the chain mediation model in this study are presented in Table 4. After incorporating emotional resilience and mindfulness into the mediation model examining the contribution of perceived social support on learning engagement, the 95% confidence intervals for both the total effect and the total indirect effect excluded zero, indicating the validity of this chain mediation model. The direct predictive path from perceived social support to learning engagement retained its significance, even after accounting for the influence of mediating variables. Specifically, the path perceived social support → emotional resilience → learning engagement showed a 95% confidence interval that did not cover zero. The analysis revealed that a substantial portion (32.64%) of the total effect of perceived social support on learning engagement was mediated by emotional resilience, a result that confirms the significance of this mediating role and thereby corroborates Hypothesis 2. The path perceived social support → mindfulness → learning engagement also showed that the 95% confidence interval excluded zero. A significant mediation effect was found for mindfulness, explaining 5.90% of the total effect from perceived social support to learning engagement. This result corroborates Hypothesis 3 (H3). Finally, the path perceived social support → emotional resilience → mindfulness → learning engagement had a 95% confidence interval excluding zero. This indicates a significant chain mediating role of emotional resilience and mindfulness in the relationship between perceived social support and learning engagement, accounting for 5.75% of the total effect, which supports Hypothesis 4 (H4).

### 3.5. Moderating Effect of Depression in Chain Mediation Model

Following the analytical steps for testing moderated mediation effects (Wen & Ye, 2014), and while controlling for gender, the moderating role of depression within the chain mediation model was examined. This test employed Model 87 from the SPSS 27.0 PROCESS macro, specifying perceived social support as the independent variable, emotional resilience and mindfulness as mediators, learning engagement as the dependent variable, and depression as the moderator.

After depression was incorporated in the chain mediation model, the mediation term of depression and mindfulness had a prominent prediction on learning engagement (β = −0.09, *p* < 0.001). This shows that there is a moderating effect of depression on the interrelationship between mindfulness and learning engagement. The specific results are presented in Table 5.

**Figure 3:**
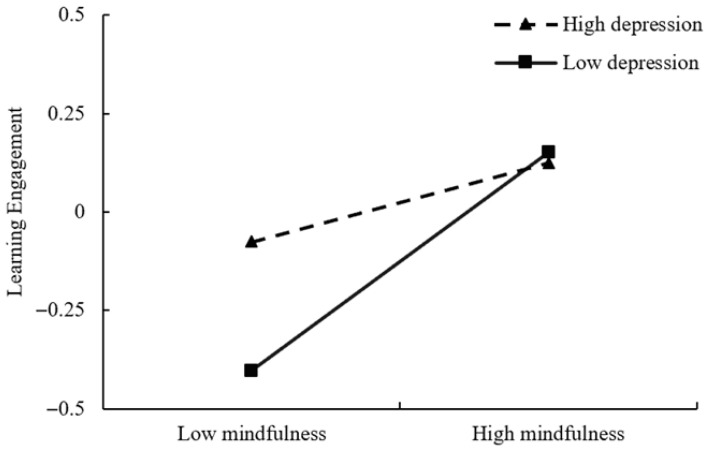
The moderating effect of depression between mindfulness and learning engagement.

To further look into the importance of this moderating effect, simple slope analysis was conducted to test whether the relationship between mindfulness and learning engagement could be varied at the different degrees of depression. The outcomes are given in Table 6 and Figure 3. Under conditions of low depression levels, mindfulness demonstrated a strong positive predictive influence on engagement in learning. Under conditions of high depression levels, this positive predictive effect of mindfulness on learning engagement was significantly attenuated, though it remained statistically significant. These results indicate that the positive influence of mindfulness on engagement in learning gradually weakens as the level of depression increases, thus suggesting that depression significantly moderates the association from mindfulness to learning engagement.

**Figure 4:**
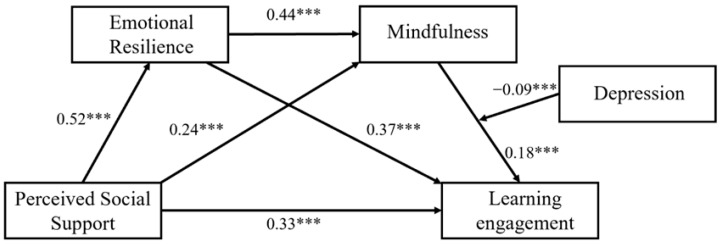
Final model path diagram. *** *p* < 0.001.

Ultimately, the path diagram for our moderated chain mediation model derived from this research is presented in Figure 4.

## 4. Discussion

According to SDT, COR, and BBT, the current study proposed and validated an integrated moderated chain mediation model, systematically exploring the impact that perceived social support has on the learning engagement of Chinese vocational school students. The results of this research did not just confirm that the perception of social support can have a direct predictive influence on learning engagement but also revealed the key psychological functions played by emotional resilience, mindfulness, and depression in this relationship. These findings indicate that in vocational education practice, attention should be paid to students’ subjective perceived support, and their engagement in learning can be encouraged by enhancing their emotional regulation abilities and the level of mindfulness.

### 4.1. Perceived Social Support and Learning Engagement

The findings provide support for H1, showing that vocational college students’ learning engagement is positively linked with increased levels of perceived social support. Importantly, even after emotional resilience and mindfulness were included in the model, perceived social support remained a significant direct predictor of learning engagement. Given that the model simultaneously controlled for two strong psychological mediators and depression, the direct path indicates that perceived social support may function as a core contextual factor of engagement rather than merely an indirect background variable. This observation is mostly similar to those made in earlier studies with comparable outcomes (Sun et al., 2023). In shaping individual learning behaviors, students who report higher levels of perceived support tend to demonstrate greater vigor, dedication, and absorption in learning activities.

This finding is consistent with international studies. In a U.S. longitudinal study, M.-T. Wang and Eccles (2012) found that teacher, peer, and parent support predicted the trajectories of multiple dimensions of school engagement from middle to high school. Similarly, a study conducted in Ghana by Ansong (2017) reported that parental and classmate support were significantly associated with emotional and behavioral engagement, although the relative strength of different support sources varied. Similar results have also been found among Asian student populations, including studies in China (Xiong et al., 2021; Zhu et al., 2025), Taiwan (Shih, 2021), and South Korea (Lee et al., 2025; J. Song et al., 2015). These findings suggest that the positive association between perceived social support and engagement is not culture-specific but appears across diverse educational systems.

In light of SDT, perceived social support assists in tapping into the basic psychological needs of individuals, especially the needs of relatedness and autonomy (Ryan & Deci, 2020). With the fulfillment of basic psychological needs among vocational college students, it is more likely that they will internalize learning as part of self-determination behavior as opposed to viewing learning tasks as externally enforced requirements. This, in its turn, elicits an intrinsic drive and promotes learning interest (Xu et al., 2024). Moreover, the cost of psychic and physical expenditures among people attempting to take up challenging activities can be held down by the existence of stable social interactions (Beckes & Coan, 2011). To an extent, the collected data serve to corroborate this, indicating that perceived social support can facilitate continued engagement in learning by fulfilling the psychological needs that are basic to people and lowering the psychological cost of learning processes. This may help vocational college students to use more resources in the case of academic challenges.

However, compared with students in comprehensive universities, vocational college students in China often face additional pressures related to employment competition and social stereotypes regarding vocational pathways. Under such conditions, perceived social support may carry heightened psychological significance. Feeling valued and understood by family members, teachers, and peers may not only provide encouragement but also buffer against perceived social marginalization. This may explain why perceived social support retained a relatively strong direct predictive effect even after accounting for internal psychological resources.

### 4.2. The Independent Mediating Roles of Emotional Resilience and Mindfulness

The findings suggested that learning engagement is facilitated by the perceived social support through boosting emotional resilience. Perceived social support allows individuals to have a psychological safe zone, thus enabling them to overcome adverse emotions fast and remain positive when faced with academic problems (Taylor, 2011). This competency to maintain a positive attitude and overcome negative emotions, that is, emotional resilience, is an important predictor of learning motivation sustainability (Galve-González et al., 2025). When students are emotionally resilient, they are rarely expected to be drawn into the depths of self-doubt and anxiety because of one failure on an exam or an academic learning problem. They are rather inclined to view difficulties through the lens of potential development, thus continuing to learn (Ge, 2025). Put differently, perceived social support, as a personal resource, assists people in amassing and safeguarding other psychological resources (including emotional resilience) to handle academic stress (Hobfoll et al., 2018), which facilitates learning engagement. H2 was thus confirmed.

The present research also established that perceived social support has the ability to affect learning engagement through an increase in the magnitude of mindfulness. Employing a caring and non-condescending atmosphere will lower the level of psychological defensiveness and anxiety in an individual, allowing them to be more receptive to the current moment, and without any concerns about it, the aspect of mindfulness can be developed (Creswell, 2017). The non-judgmental awareness characteristic of mindfulness that enables non-focus prevents students from gaining retrospective regrets and future anxiety concerns but helps them focus on the present learning activity (Brown & Ryan, 2003; Dundas et al., 2016). This state of concentration in itself constitutes a foundational component that defines active learning engagement (Schaufeli, 2013). Thus, perceived social support, through the establishment of a safe psychological space, makes it possible to develop mindfulness, which, in turn, brings about an increase in the depth and persistence of learning. H3 was thus confirmed.

### 4.3. The Chain Mediating Role of Emotional Resilience and Mindfulness

This study validated a sequential mediating aspect of emotional resilience and mindfulness against perceived social support and learning engagement. An analysis of the data reveals that there is a complex process of psychological mechanisms: Perceived social support initially strengthens the emotional resilience of a person. This increased emotional resilience consequently leads to the positive grounds of the development of mindfulness, which eventually enhances learning engagement. This pattern aligns with findings from other cultural contexts, though the specific mechanisms have rarely been tested as a sequential chain. Research in Western samples has separately documented the links between perceived social support and resilience (Turner et al., 2017; Yalcin-Siedentopf et al., 2021), resilience and mindfulness (Garland et al., 2015a), and mindfulness and engagement (Bamber & Schneider, 2022; Coo & Salanova, 2018). The present study extends this literature by demonstrating that these processes operate sequentially within a single integrated model.

This conclusion unites the COR and BBT. Perceived social support is the initial resource input, and it assists one in safeguarding and cementing their emotion regulation capacity (emotional resilience). Emotional resilience allows a person to possess more mental energy to increase their cognitive and attentional flexibility and not to use it to deal with negative emotions (Fredrickson, 2001). This improved cognitive elasticity is one of the requirements of developing mindfulness, that is, having the capacity to anchor one’s attention in the present and perceive it neutrally and without prejudice (Garland et al., 2015b). Altogether, H4 is confirmed as it is shown that emotional resilience and mindfulness sequentially mediate the bearing of perceived social support on the learning engagement of vocational students.

### 4.4. The Moderating Role of Depression

Moderation was observed in terms of the variable of depression between mindfulness and learning engagement, a fact that validates H5. In particular, the aspect of the promotive effect of mindfulness on learning involvement was stronger at lower levels of depression but much weaker at higher levels of depression. Depressive conditions usually consist of anhedonia and a lack of motivation that erodes the sense of worth and control of an individual in learning processes (Gotlib & Joormann, 2010; Pekrun, 2006). Under high levels of depression, students might feel that learning is a meaningless or unachievable task. Although they might be able to strengthen their concentration in the short term with the help of mindfulness practice, the said focus might not easily transfer into long-term and ardent learning activity.

Additionally, though the positive influence of mindfulness on learning engagement was reduced somewhat, it did not disappear entirely, even at high levels of depression. This suggests that mindfulness, as a strategy for attention and emotion regulation, retains a certain protective significance for students experiencing depression. This means that mindfulness as an attention and emotion regulation strategy still has an element of a protective role in depressed students (Ayhan & Kavak Budak, 2021). It might assist students in being slightly disengaged from negative thinking rumination so that they can use their limited cognitive resources to study. The findings of this research suggested that mindfulness training might not be adequate when it comes to managing the learning difficulties of groups with high levels of depression in the context of undertaking psychological interventions in vocational college students. In order to ensure an optimal contribution of the promotive effect on the engagement of learning, the combination of mindfulness training and solutions to the primary symptoms of depression, including cognitive behavioral therapy or drug use, may represent a more efficient approach.

### 4.5. Implications

There are important implications of the discoveries of this research study on pedagogical practice, interventions in mental health, and the integrative growth of students with respect to families, schools, and society in vocational colleges. Vocational colleges, on the one hand, should methodically build and actively develop a supportive campus environment. School administrators and teachers should take the initiative to attend to the mental needs of students by forming active relationships between themselves and pupils, promoting peer support, and offering students’ academic and career guidance on time. By doing so, this strategy may help students to feel more supported and cared for by the school, and thus their needs regarding relatedness and autonomy will be initially satisfied, as well as triggering their intrinsic drive to learn. Moreover, since emotional resilience and mindfulness have a critical mediation role in facilitating learning engagement, schools can introduce modules on emotion regulation, stress management, and mindfulness meditation into mental health education curricula or general education courses. They can equip students with skills to overcome negative emotions and maintain present-moment awareness by making use of workshops and group counseling sessions in order to be better able to transfer perceived social support into enduring learning motivation. Nevertheless, among more seriously depressed students, solely relying on the addition of improved mindfulness or social support may not be effective enough to drive the ability to engage in learning. Hence, educators must specify a hierarchy of interventions and offer more specific approaches to intervention. In the case of the usual student population, the universal training of mindfulness and emotion control can be introduced. In the case of students who are screened with mild to moderate symptoms of depression, intensive programs that combine mindfulness training with depression-specific intervention (e.g., behavioral activation, cognitive restructuring) need to be provided, paying particular attention to rebuilding their sense of connectedness and their perception that others genuinely care. In the case of students who have severe depression, they must be promptly referred to professional psychotherapy or medical institutions to facilitate their primary clinical treatment.

Family, on the other hand, is an extremely important perceived social source among vocational college students. It is time that parents change their views on education and provide their children with more emotional support and approval, as opposed to emphasizing results only. The key is not simply to provide support but to do so in ways that children can recognize and internalize—through expressions of affection, active listening, and non-judgmental acceptance. Research from East Asian contexts suggests that perceived parental emotional support is particularly beneficial for adolescents’ motivation and achievement. Media sources and businesses at the societal level will be advised to actively ensure the value of vocational education is promoted, which will result in the creation of a social environment focusing on the necessity to master a skill. This will increase the professional self-concept and social respect of vocational students. Such societal-level efforts can enhance vocational students’ perception that they are valued members of society, contributing to their overall sense of being supported.

## 5. Limitations and Future Directions

This research utilized the cross-sectional design methodology as it investigated the association between perceived social support and engagement in the learning of Chinese vocational students. It is therefore hard to make a rigid causal conclusion about the correlations between the variables. Research studies in the future can take the form of longitudinal follow-ups or controlled trials that can help in more conclusively testing the causal orientation underlying the model. And although the measurement model showed acceptable fit based on the RMSEA, the CFI and TLI fell slightly below conventional thresholds. This suggests that the factor structure of the measures, while adequate for the main analyses, may not be fully optimal. Future research could explore alternative factor structures or use shorter scale versions to reduce model complexity and potentially improve incremental fit indices. Moreover, the sample of this research was arranged in the vocational colleges of one region, which can make converting the findings to the other regions and dissimilar sorts of establishments uncertain. Given that vocational education systems vary across countries and that cultural factors may shape how social support is perceived and enacted, cross-cultural comparisons would help determine the generalizability of the proposed mechanisms.

Beyond these methodological considerations, the findings themselves point to several questions that warrant further investigation. The chain mediation effect of emotional resilience and mindfulness suggests that these two psychological resources operate sequentially in translating perceived social support into learning engagement. However, the data of this study cannot reveal how this sequential process unfolds over time—whether emotional resilience consistently precedes mindfulness or whether a reciprocal relationship exists between them. Longitudinal research could clarify the temporal dynamics of this chain mediation. Similarly, while the findings show that higher depression levels weaken the positive effect of mindfulness on engagement, they do not indicate whether targeted interventions could restore this effect or what types of support might be the most beneficial for students with elevated depressive symptoms. Future studies could examine whether combining mindfulness training with depression-focused strategies yields stronger effects for this subgroup. Also, the overall model was tested within a single population—Chinese vocational college students. Finally, when we discussed the influence on learning engagement, the scope of analysis was confined to the perceptions of available social support. In future studies, researchers could use objective social support to delve deeper into the interactive effects of subjective and objective support on vocational college students.

## 6. Conclusions

This study, focusing on Chinese vocational college students, constructed and tested a moderated chain mediation model. This model indicates that perceived social support directly predicts their learning engagement; meanwhile, the independent and intervening effects of emotional resilience and mindfulness indirectly affect their engagement in learning. Additionally, depression is a moderating factor in the pathway of mindfulness and engagement in learning. However, this pathway is conditional: its effectiveness depends on students’ mental health status. For those with elevated depressive symptoms, the protective function of mindfulness is diminished, suggesting that mindfulness-based interventions alone may be insufficient for this subgroup.

These findings matter for vocational education because they identify emotional resilience and mindfulness as precise psychological targets for intervention that educators can strengthen to help students translate perceived support into sustained engagement. They also underscore the importance of addressing depressive symptoms to ensure that such efforts are effective. This research offers fresh empirical data on the learning behaviors among this population and offers a theoretical foundation and practical implications that vocational colleges can use in providing learning support and psychological interventions. By attending to both the promotion of psychological resources and the mitigation of mental health challenges, vocational institutions can more effectively support students’ academic success and personal well-being.

## Figures and Tables

**Table 1:** Demographics of participants.

Variables	Form	Frequency	Percent
Gender	Man	614	39.7%
	Woman	913	60.3%
Grade	First Year	826	53.5%
	Second Year	354	22.9%
	Third Year	365	23.6%
Academic Major	Science and Engineering	824	63.3%
	Humanities and Social Sciences	165	10.7%
	Agriculture	17	1.1%
	Arts and Physical Education	131	8.5%
	Others	408	26.4
Place of Origin	Urban	567	36.7%
	Rural	978	63.3%
Only Child	Yes	258	16.7%
	No	1287	83.3%

**Table 2:** Descriptive statistical correlations analysis results (*N* = 1545).

Variable	M	SD	Max	Min	1	2	3	4	5
1. Perceived Social Support	5.04	1.05	1	7	1				
2. Emotional Resilience	3.75	0.85	1	6	0.52 ***	1			
3. Mindfulness	4.55	0.81	1	6	0.46 ***	0.56 ***	1		
4. Depression	2.06	0.72	1	4	−0.45 ***	−0.62 ***	−0.57 ***	1	
5. Learning Engagement	3.77	1.20	1	7	0.57 ***	0.60 ***	0.49 ***	−0.40 ***	1

Note: M = mean; SD = standard deviation. *** *p* < 0.001.

**Table 3:** Regression analysis in the chained mediation model.

Regression Equation		Overall Fitting Index			Significance of Regression Coefficient		
Outcome Variable	Predictor(s)	R	R^2^	F	β	t	*p*
Emotional Resilience	Gender	0.53	0.28	298.78 ***	0.11	5.27 ***	<0.001
	Perceived Social Support				0.52	23.94 ***	<0.001
Mindfulness	Gender	0.60	0.36	286.30 ***	−0.02	−1.12	0.2650
	Perceived Social Support				0.24	9.83 ***	<0.001
	Emotional Resilience				0.44	18.44 ***	<0.001
Learning Engagement	Gender	0.68	0.46	331.29 ***	0.01	0.45	0.6560
	Perceived Social Support				0.32	13.98 ***	<0.001
	Emotional Resilience				0.36	14.69 ***	<0.001
	Mindfulness				0.14	6.09 ***	<0.001

Note: *** *p* < 0.001.

**Table 4:** Bootstrap test for chain mediation effects.

Path	Effect Value	BootSE	BootLLCI	BootULCI	Ratio
Total Effect	0.5661	0.0210	0.5250	0.6072	
Direct Effect	0.3153	0.0226	0.2711	0.3596	55.70%
Total Indirect Effect	0.2508	0.0196	0.2137	0.2900	44.30%
X → M1 → Y	0.1848	0.0194	0.1479	0.2238	32.64%
X → M2 → Y	0.0334	0.0078	0.0191	0.0496	5.90%
X → M1 → M2 → Y	0.0326	0.0064	0.0191	0.2238	5.76%

**Table 5:** Moderated mediation effect of different levels of depression in chain mediation model.

Regression Equation		Overall Fitting Index			Significance of the Regression Coefficient	
Outcome Variable	Predictor(s)	R	R^2^	F	β	t
Learning Engagement	Gender				0.01	0.17
	Perceived Social Support				0.33	14.70 ***
	Emotional Resilience	0.69	0.48	234.19 ***	0.37	14.32 ***
	Mindfulness				0.19	7.71 ***
	Depression				0.07	2.95 **
	Mindfulness × Depression				−0.09	−5.83 ***

Note: ** *p* < 0.01, *** *p* < 0.001.

**Table 6:** Moderated mediation effect of different levels of depression in mindfulness and learning engagement.

	Effect Value	BootSE	BootLLCI	BootULCI
M − 1SD	0.2778	0.0309	0.2172	0.3384
M	0.1893	0.0246	0.1411	0.2375
M + 1SD	0.1008	0.0267	0.0485	0.1531

## Data Availability

The data are available from the corresponding author upon reasonable request.
